# Pluripotential Risk and Clinical Staging: Theoretical Considerations and Preliminary Data From a Transdiagnostic Risk Identification Approach

**DOI:** 10.3389/fpsyt.2020.553578

**Published:** 2021-01-08

**Authors:** Jessica A. Hartmann, Patrick D. McGorry, Louise Destree, G. Paul Amminger, Andrew M. Chanen, Christopher G. Davey, Rachid Ghieh, Andrea Polari, Aswin Ratheesh, Hok Pan Yuen, Barnaby Nelson

**Affiliations:** ^1^Orygen, Parkville, VIC, Australia; ^2^Centre for Youth Mental Health, The University of Melbourne, Parkville, VIC, Australia; ^3^BrainPark, The Turner Institute for Brain and Mental Health, School of Psychological Sciences and Monash Biomedical Imaging Facility, Monash University, Melbourne, VIC, Australia; ^4^Department of Psychiatry, The University of Melbourne, Parkville, VIC, Australia

**Keywords:** at-risk (youth), transdiagnostic, pluripotency, clinical staging model, at risk mental state

## Abstract

Most psychiatric disorders develop during adolescence and young adulthood and are preceded by a phase during which attenuated or episodic symptoms and functional decline are apparent. The introduction of the ultra-high risk (UHR) criteria two decades ago created a new framework for identification of risk and for pre-emptive psychiatry, focusing on first episode psychosis as an outcome. Research in this paradigm demonstrated the comorbid, diffuse nature of emerging psychopathology and a high degree of developmental heterotopy, suggesting the need to adopt a broader, more agnostic approach to risk identification. Guided by the principles of clinical staging, we introduce the concept of a pluripotent at-risk mental state. The clinical high at risk mental state (CHARMS) approach broadens identification of risk beyond psychosis, encompassing multiple exit syndromes such as mania, severe depression, and personality disorder. It does not diagnostically differentiate the early stages of psychopathology, but adopts a “pluripotent” approach, allowing for overlapping and heterotypic trajectories and enabling the identification of both transdiagnostic and specific risk factors. As CHARMS is developed within the framework of clinical staging, clinical utility is maximized by acknowledging the dimensional nature of clinical phenotypes, while retaining thresholds for introducing specific interventions. Preliminary data from our ongoing CHARMS cohort study (*N* = 114) show that 34% of young people who completed the 12-month follow-up assessment (*N* = 78) transitioned from Stage 1b (attenuated syndrome) to Stage 2 (full disorder). While not without limitations, this broader risk identification approach might ultimately allow reliable, transdiagnostic identification of young people in the early stages of severe mental illness, presenting further opportunities for targeted early intervention and prevention strategies.

## Introduction

Over the past decade, we have observed increased public awareness of the prevalence and debilitating consequences of severe mental illness. A substantial contributor to the burden of severe mental illness can be the long, progressive illness trajectories that typically become established early in a person's life, generally during adolescence, or late childhood (1, 2). Consequently, there has been a move toward early identification and intervention frameworks, with the aim of reducing the burden by halting the progression of illness or preventing the onset of disorder altogether (3–5).

However, a successful move toward earlier identification and intervention requires a different operationalisation of psychopathology than its current, increasingly criticized, form. Current diagnostic and research systems are less appropriate for these early approaches, as they are based on cross-sectional features observed in entrenched or chronic mental illness, thus embodying the “end-state” of illness trajectories only, failing to represent the progressive and dynamic nature of (emerging) psychopathology (6, 7). Analogically speaking, this corresponds to relying on descriptions of cancer based on final stages of the disease only, ignoring any earlier cell anomalies and their progressive dynamics which might have been present for years. Furthermore, the staggering extent of co-morbidity (8, 9) and phenomena present across disease entities, such as psychomotor slowing, agitation, anhedonia, or delusions, especially early in illness trajectories (10–14), do not support the status quo of separate diagnostic classes. Similarly, a rapidly emerging body of research investigating interacting symptom networks demonstrates widespread and significant interconnections between different diagnostic entities (15–17).

Despite the barriers caused by the current operationalization of mental illness, researchers in Australia initiated a significant move toward pre-emptive psychiatry and early intervention by developing the ultra-high risk (UHR) criteria two decades ago (18). The clinical criteria, identifying young people at risk of developing first episode psychosis (FEP) by a combination of attenuated/short-lived psychotic symptoms and/or trait vulnerability^1^, did not rely on thresholds provided by current diagnostic systems. The UHR paradigm was developed based on the long-standing understanding that psychotic disorders do not emerge “out of the blue,” but typically have a forerunner phase characterized by milder symptomatology and functional decline (18, 20, 21). This paved the way for a clinical staging model as further outlined below. Three decades later, a multitude of studies have shown that these criteria have a valence for psychosis, *as well as for other disorders* (mainly anxiety and depression) (22–24), further challenging the siloed approach to diagnostic systems. Similarly, only a small proportion of young people in FEP programs linked to UHR programs go through UHR clinics first (25), implying that there might be alternative early symptom trajectories leading to psychosis, which might be missed by services focusing exclusively on (attenuated) psychotic symptoms (21). These observations, and the modest proportion of UHR young people transitioning to psychosis in research trials [~20% over 2 years (26)] causing statistical challenges for the design of intervention studies (27), highlight the need for the development of a broader, transdiagnostic at-risk approach.

The great challenge lies in developing these wider, transdiagnostic frameworks for early risk identification while maximizing *clinical utility*. Guided by the principles of the clinical staging framework, we will introduce the concept of a pluripotent at-risk mental state. First, we will provide a theoretical overview of the underpinnings of clinical staging and pluripotency; second, we will present the Clinical High At Risk Mental State (CHARMS) approach and some preliminary data of this ongoing cohort study.

## Clinical Staging

“Sub-threshold” versions of mental disorders (i.e., conditions that fall below the threshold of “caseness” as defined by psychiatric diagnostic systems), frequently precede later full syndrome disorders (28), aligning with the increasingly prevailing notion that there is no clear-cut demarcation between absence and presence of mental disorder (29, 30). Dimensional models of mental disorder conceptualize psychopathology on a continuum of severity, ranging from mild liability or expression at one end of the spectrum to fully-fledged, chronic and treatment-resistant disease at the other (31–34). While the view of a continuum of illness and illness progression has gained traction, it poses a challenge for the process of clinical decision making and clinical communication, which inherently requires thresholds (35, 36). Clinical staging, a framework adapted from other areas of medicine, adopts the dimensional approach to mental illness while offering step-wise anchors for phase-specific treatment selection (6, 37, 38). In other words, a person's clinical presentation is mapped onto the spectrum of mental illness, informing intervention plans, and offering a prognosis of potential trajectories of progression and remission (5). In clinical practice, this translates into less aggressive, safer, and more targeted treatment approaches. Rather, a staged care approach recognizes the need for interventions that are tailored according to symptom severity, allowing clinicians to provide low-intensity interventions for patients with milder presentations along the spectrum of illness, prior to reaching a full-threshold disorder.

Stages are defined using symptom severity, specificity, persistence and disability. An early stage is typified by mild symptom severity, a lack of specificity, mild functional impairment; an advanced stage is associated with severe symptom burden, clearer syndromal specificity and stability, significant functional impairment and persistent/recurrent patterns (39, 40). The original clinical staging model spans from stage 0 to stage 4, starting with an at-risk but asymptomatic state (Stage 0) and increases in severity to help seeking, nonspecific symptoms (Stage 1a), attenuated syndrome (stage 1b), full-threshold disorder (Stage 2), recurrence and persistence of illness (Stage 3), and lastly severe and chronic mental health disorders (Stage 4). A revised version of the clinical staging model, currently only focused on psychosis, has been formulated (41). This model further segregates Stage 1 (“high-risk”) into three sub-stages with increasing symptomatic specificity, moving from negative/cognitive symptoms (1a) and attenuated symptoms (1b) to short lived remitting episodes (1c). Stage 3 (“late/incomplete recovery”) is also further subdivided into three stages, moving from single relapse (3a) over multiple relapses (3b) to incomplete recovery from first episode (3c).

A key assumption of the clinical staging framework is that a return to previous stages is not possible. For example, a client in Stage 3a can fully remit, i.e., a “Stage 3 in full remission”; however, they cannot return to a “Stage 0” nor to “Stage 1.” In fact, remission and recovery is an integral part of the staging process. Stage 2 is associated with symptomatic and functional early full recovery (remission). Stage 3 is associated with late/incomplete recovery in any symptomatic or functional domain while Stage 4 is severe, persistent, or unremitted illness (41). Recent research has shown that 20% of individuals identified to be at Stage 1b progressed to a more severe stage within 12 months (42). Interrater reliability for the clinical staging model has been found to be adequate, with 90% concordance between independent raters (*k* = 0.72) (39). Cross et al. (43) identified a range of variables in transdiagnostic samples with attenuated symptoms (Stage 1b) which were associated with progression to a full-threshold disorder (Stage 2) such as not being in education, being unemployed and greater negative symptom severity (43).

The aim is to further develop clinical staging into a clinicopathological framework, linking clinical features with objective pathophysiological measures, improving precision of intervention and prognosis (6). Most importantly, it is a diagnostic framework that increases clinical utility.

## A Pluripotential at Risk Mental State: CHARMS

The shortcomings of current diagnostic classification systems, new findings regarding the dynamic and overlapping nature of psychopathology and its heterotypic trajectories, including lessons learned from the UHR paradigm regarding specificity and predictive values, all indicate that we need a new, less siloed, and early risk identification approach. This led to the development of the CHARMS (Clinical High At Risk Mental State) identification strategy, a pluripotential at-risk mental state which broadens both inputs and outputs beyond psychosis and maximizes clinical utility by building on the clinical staging framework (5, 21, 44–47). Currently, we are in the process of conducting a pilot study validating and further refining the “CHARMS criteria.” The CHARMS criteria are a set of clinical criteria that define a pluripotential at risk mental stage as described above and capture risk for a *range* of different outcomes (see below). Operationally, the CHARMS criteria are a broadening of the existing UHR criteria, extending it from UHR (capturing subthreshold versions/genetic vulnerability for psychotic disorder) to capturing subthreshold versions/genetic vulnerability for affective (unipolar and bipolar depression) and borderline personality disorder (BPD). The decision to also include BPD was informed by evidence that young people with emerging BPD features show non-specific and evolving mixtures of signs and symptoms that substantially overlap with precursors of bipolar and psychotic disorder, recognizing that the early stages of these disorders cannot yet be disentangled adequately to support disorder-specific identification frameworks and preventative interventions (48–50).

CHARMS is an extension of the UHR state and represents the clinical operationalisation of the first stage requiring significant clinical attention in the clinical staging model, that is, Stage 1b. Therefore, recruitment into the CHARMS cohort study is based on *presentation to services* (i.e., headspace centers in metropolitan Melbourne, Orygen Specialist Clinical Services) rather than based on presence of specific diagnoses.

### The Term Pluripotency

The idea of a “pluripotential at risk mental state,” introduced for the first time by Johannessen and McGorry (51) has attracted criticism and misconceptions based on the term used. First, there has been a perception in the literature that the term “pluripotent risk” refers to the existing UHR operationalisation, i.e., the *UHR for psychosis state* itself is considered pluripotent in that it predicts the onset of disorders other than psychosis (52–55). While UHR is indeed part of CHARMS, the idea of a pluripotential at-risk mental state was always to broaden both input and output points, thus moving beyond UHR for psychosis and considering other mental disorders (44). Second, the term “pluripotential” should be interpreted in light of the clinical staging framework, rather than through the lens of cell biology or oncology as some critics have done (56). Literally, “pluri” refers to *several*, “potential” refers to *capable or possible*: It reflects the potential for the picture to evolve into several syndromes or outcomes (51), akin to heterotypy. In other words, we remain agnostic about the future trajectory of the disorder, and simply maintain that a broad range of outcomes are possible. This is not to say that all mental illness is a manifestation of the exact same origin, and the specific trajectory is a result of environmental influences (57). As an example, we do not assert that *every* young person meeting CHARMS criteria, regardless of clinical presentation and genetic make-up, placed in a certain environment, is capable of developing a particular syndrome. Rather, the CHARMS criteria aim to identify young people who are presenting with unspecified, sub-threshold levels symptoms consistent with stage 1b of the clinical staging model, and therefore considered to be a population with a high risk of transition to a range of full threshold disorders.

### Specificity Might Increase Over Time

Among the core principles of CHARMS is the assumption that psychopathology in its earliest stages is protean and non-specific, with greater specificity for certain disorders gained in later stages (58). This is not too dissimilar from the view of a general “liability” psychopathology factor crystallizing into more specific conditions with increasing age (“p-differentiation”) (59–61). While the empirical evidence for p-differentiation has been mixed, these studies have investigated differentiation as a function of age rather than illness severity as suggested by the clinical staging framework.

Another source of support for later, more stable symptom patterns stems from studies investigating the network structure of psychopathology. These studies show that there is increasing connectivity in symptom networks with increasing levels of severity (62–65), pointing toward increasing stability in psychopathology. While a recent study failed to demonstrate increase in global network structure with increasing severity (66), network findings are generally in line with large epidemiological studies. These epidemiological studies have shown how non-specific states in children and adolescents, usually characterized by anxiety and depressive symptoms, develop into more stable adult-type major mood (depression, bipolar) or psychotic disorders (11, 67–69). For this reason, CHARMS proposes a transdiagnostic “lumping” approach (i.e., not differentiating between diagnostic entities as defined by international diagnostic symptoms), representing the most useful approach for providing healthcare to young people with undifferentiated clinical representations. By doing so, it also provides a sampling frame for prospectively researching the evolution of early stages of severe mental disorder, which has been hampered to date by the “diagnostic silo” approach to risk identification. Furthermore, it aligns with studies demonstrating the shared genetic and neurobiological basis of mental illness, as well as the number of shared environmental risk factors (70–73), as further outlined below.

### Transdiagnostic

The *transdiagnostic approach* “involves trying to understand the shared, overarching processes that cut across the classification system” [(74), p. 360]. Although suggested otherwise by some authors (75, 76), the clinical staging framework adopts a transdiagnostic approach. This is in line with the “Research Domain Criteria” (RDoC) introduced by the National Institute of Mental Health (NIMH) (77–79) and the Hierarchical Taxonomy of Psychopathology (HiTOP) (80, 81).

RDoC aims to “implement, for research purposes, a classification system based upon dimensions of observable behavior and neurobiological measures” (78). In other words, the biopsychological basis of fundamental psychological constructs (e.g., reward seeking, memory, and fear) are explored and linked to clinical phenotypes (79). RDoC has primarily been developed as a research tool, not as an aid for practical clinical decision making. It aims to find (biological) explanations for clinical problems and to *inform* clinical schemes, such as the clinical staging framework. It also does not incorporate the temporal or dynamic aspect and does not facilitate early identification purposes. Thus, RDoC represents a framework *complementing* CHARMS and clinical staging, rather than representing a competing framework.

Similar to RDoC, HiTOP represents a hierarchical, dimensional approach to psychopathology, with lower-level syndromes based on empirical covariation of signs and symptoms which form higher-level spectra based on covariation of syndromes (80–82). This idea is related to the concept of micro-and macro phenotypes first articulated by van Os (83). In the context of HiTOP, the p-factor can be seen as very broad “super-spectrum,” representing features shared across all mental disorders (81). As stated above, the p-factor and CHARMS approach have the same underlying idea of an underlying vulnerability for psychopathology that is not differentiated by disorder. However, the p-factor describes a transdiagnostic *structure* (i.e., a factor which is present across disorders). In contrast, the term “transdiagnostic” in CHARMS does not necessarily refer to a common shared factor, but rather to *not differentiating* or *not separating into diagnostic silos* according to clinical presentation.

Similar to HiTOP, aiming to integrate the traditionally separate domains of personality and psychopathology (84, 85), the CHARMS approach also aims to bridge the traditional separation between personality and psychopathology. The CHARMS criteria include borderline personality pathology, as this represents a general severity factor in personality pathology (86) and because subthreshold borderline pathology is clinically significant in young people (87).

The transdiagnostic approach in CHARMS has been criticized for pooling together potentially different phenotypes and illness trajectories, thereby interfering with individual risk prediction and specific treatment development (88). However, the CHARMS approach does not prohibit the identification of specific illness trajectories or risk factors for specific phenotypes (see below).

### Homotypic vs. Heterotypic Continuity of Psychopathology

There is evidence that young people at UHR for psychosis also have incident or persistent disorders other than psychosis (22–24). Similarly, there is evidence that young people at risk for non-psychotic disorders (such as depression) might develop psychotic disorders (54). Both these trajectories are examples of heterotypic development, i.e., one condition predicting another condition at a later time point (89) There is increasing evidence for heterotypic continuity in children and adolescents (14, 90, 91). For example, a recent study in the ALSPAC cohort (*N* = 4,815, ages 7.5–14 years) demonstrated widespread heterotypic continuity, even when controlling for homotypic continuities (92). However, heterotypic continuity is also observed in “established” disorder (9, 93). A recent Danish registry study (*N* = 5,940,778) showed that any given index mental illness is associated with an increased risk of developing any other mental illness, even across diagnostic class (94). Similarly, the Dunedin Study birth cohort (*N* = 1,037) demonstrated that mental disorder life history traverse across internalizing, externalizing, and thought disorders and all disorders are associated with an increased risk for all other disorders (95). These studies support the idea that mental disorder categories are not a static. Rather, they are highly dynamic process that ought to be considered from a developmental perspective. It also provides support for the idea of pluripotentiality, i.e., it might be useful to not specify the “terminus” of an illness trajectory. The clinical staging/CHARMS approach allows for both (capturing of) homotypic progression (e.g., young person with depressive symptoms without significant comorbidities goes on to develop recurrent depression) and heterotypic progression (e.g., young person with depressive and attenuated psychotic symptoms goes on to develop first episode mania). This will allow one to investigate and specify stable (homotypic, transdiagnostic) continuity, as well as heterotypic, disorder specific continuity (96).

### The Power Problem and Prevention Paradox

By widening the outcome target and including high-prevalence disorders such as depression, we not only allow for heterotypic development on a conceptual level, but also allow for greater statistical power at a methodological level. As originally noted by Cuijpers (27), prevention trials rarely investigate whether they are, in fact, able to reduce the incidence of the disorder in question, as the number of participants needed for this is high, especially if the incidence of the target syndrome is low. This is partly explained by the relative non-specificity of known risk factors, as discussed above. By increasing the incidence rate of the target outcome in the population, we drastically reduce the number of participants needed to achieve adequate statistical power. The CHARMS approach increases the incidence rate of new disorders by following all three recommendations by Cuijpers (27): (1) focusing on indicated prevention (symptoms are present without reaching “full threshold”), (2) focusing on high-risk groups with multiple risk factors, and (3) focusing on target groups with multiple disorders. This approach also addresses the “prevention paradox” and “relative blindness” raised in traditional UHR research (45, 97, 98).

### The CHARMS Study: Study Details and Preliminary Findings

As mentioned, we are in the process of conducting a pilot study validating and further refining the “CHARMS criteria.” The CHARMS criteria can be sub-divided into four at-risk mental states: high risk for psychosis (UHR), high risk for severe depression (HRD), high risk for mania (HRM), and high risk for borderline personality disorder (HRB), although this division is not the focus of the study. Guided by the UHR criteria, the CHARMS criteria are based on subthreshold symptoms (comprising attenuated psychotic, moderate depressive, subthreshold manic symptoms, and BPD features), trait vulnerability and functional decline. For a detailed description of the criteria, see Hartmann et al. (46). As the focus of this study is on the (broad) early clinical phase of illness, all clients of our clinical services are eligible for the study regardless their presentation, unless full-threshold for illness (the main outcome of the study) has already been reached. That is, inclusion comprises one criterion: Help-seeking at our clinical services. Exclusion criterion comprises ≥ Stage 2 of illness. Important for recruitment, and key to the underlying CHARMS risk identification approach, are our *headspace* enhanced primary care services. *headspace* represents a transdiagnostic early intervention service for young people aged 12–25 with a range of subthreshold and threshold presentations and is the main recruitment source for the CHARMS study. Recruitment also takes place at Orygen Specialist programs (a secondary mental health service), however given our exclusion criterion of ≥Stage 2 and the more severe clinical presentation at the specialist programs compared to headspace, this is a minor source.

Consenting participants meeting the CHARMS criteria at baseline are allocated to *CHARMS***+** (i.e., Stage 1b). Those falling below threshold are allocated to *CHARMS*– (i.e., Stage 1a, the control group). Participants meeting criteria for Stage 2 (for example, first episode psychosis or mania) are excluded. Participants are re-assessed after 6 and 12 months.

**Figure 1:**
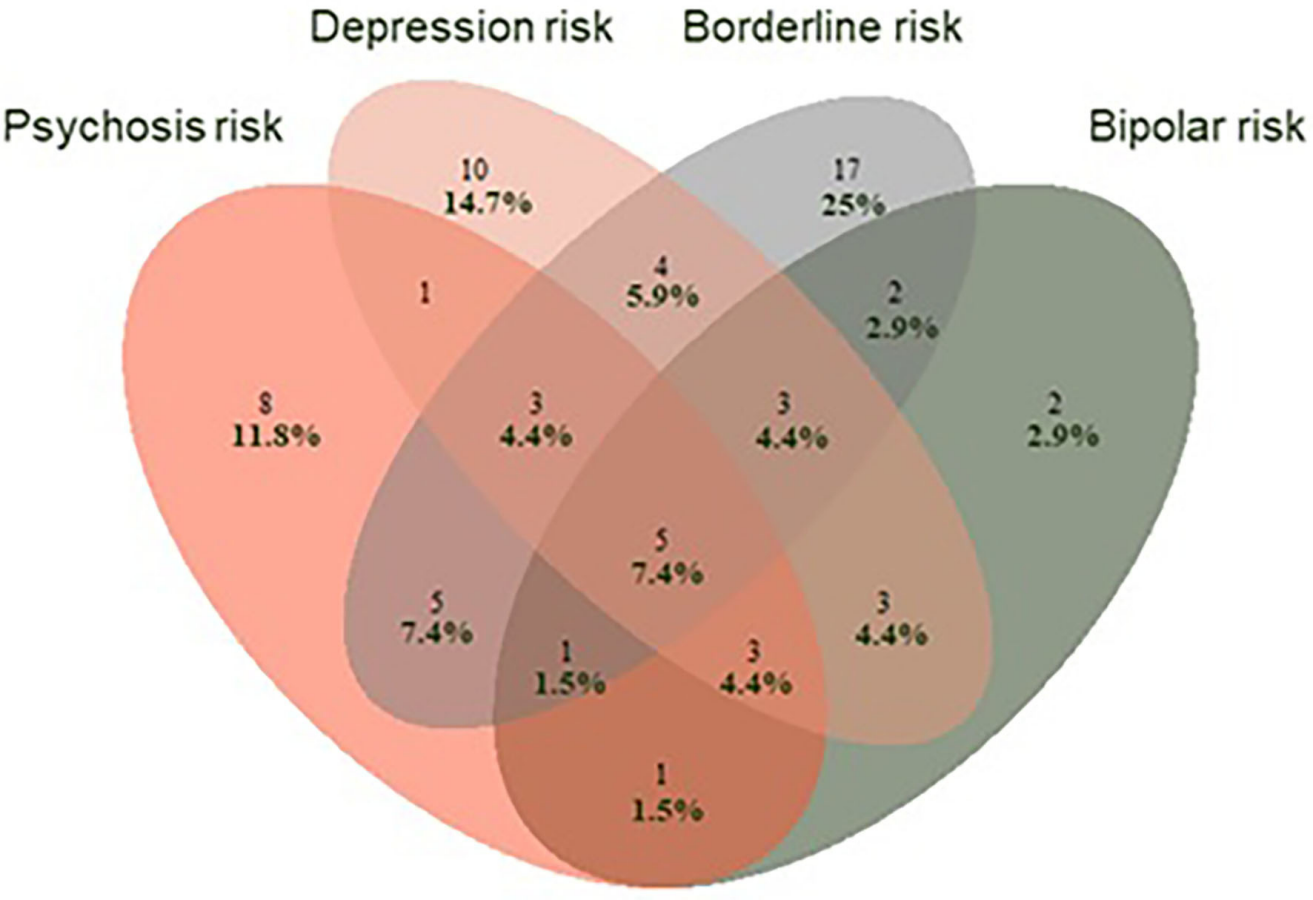
Venn Diagram showing the extent of overlap of the four at-risk groups at baseline for those that meet CHARMS criteria (*N* = 68) at baseline.

**Figure 2:**
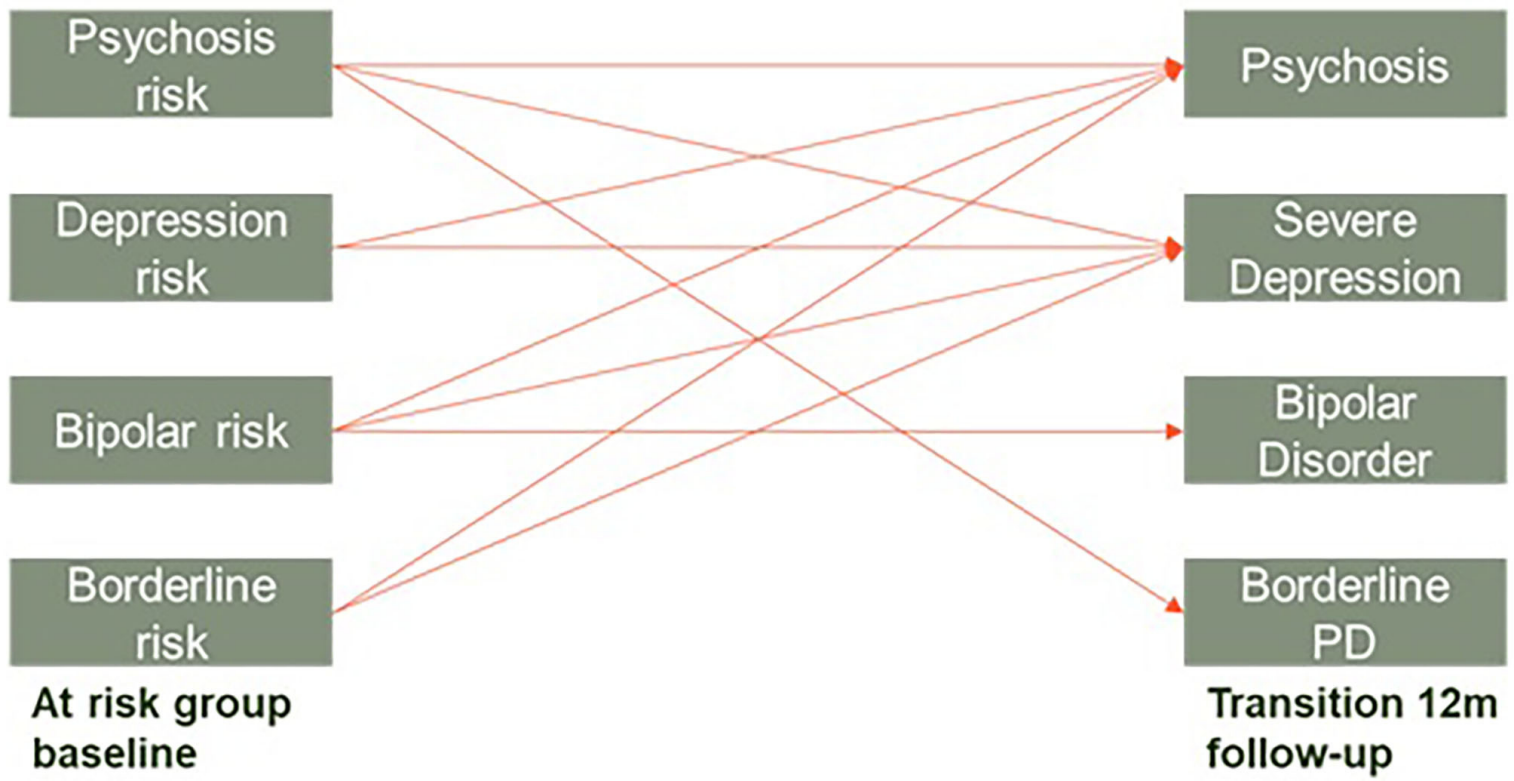
Observed homotypic and heterotypic continuity from baseline to 12-months follow-up in those meeting CHARMS criteria. Young people can meet for multiple at risk groups.

Our preliminary results (sample *N* = 114, ongoing recruitment) support the CHARMS concept. Sixty-eight percent of participants (68%) met CHARMS criteria at baseline and were allocated to the CHARMS+ group with the remainder allocated to the CHARMS– control group (please see Table 1 for an overview of clinical and demographic variables). Of the CHARMS+ group, almost half (46%) satisfied the criteria for more than one at-risk group. Figure 1 demonstrates the extensive overlap between the four different at-risk mental states. Of those who have completed the month 12 assessment in the CHARMS+ group thus far (*N* = 78), 34% have transitioned to a Stage 2 disorder (mostly severe depression), compared with 3% in the CHARMS- group. Survival analysis (Kaplan Meier) on these preliminary data show a significant difference in these transition rates between the two groups (*p* = 0.004). Interestingly, the risk for transition (by 12 months) to Stage 2 in the CHARMS+ group increases to 40% if three or more at risk states are met. When we investigate the patterns of transition, we see homotypic (e.g., a young person meeting high-risk for psychosis transitions to first-episode psychosis) as well as heterotypic development [e.g., a young person meeting high-risk for psychosis transitions to severe depression (Figure 2)], further supporting the CHARMS approach.

**Table 1:** Preliminary baseline characteristics.

	**Total**	**CHARMS+**	**CHARMS–**	*P*-value^*^
*N*	114	68	46	
Age (SD)	19.62 (3.49)	19.75 (2.89)	19.43 (4.29)	n.s.
Female (%)	71 (62%)	41 (60%)	30 (65%)	n.s.
In full or part-time education (%)	76 (67%)	40 (59%)	36 (78%)	0.03
Full- or part-time employed (%)	46 (40%)	26 (38%)	20 (43%)	n.s.
SOFAS (SD)	63.95 (13.49)	60.90 (13.29)	68.72 (12.51)	0.002
QIDS (SD)	7.17 (4.38)	8.75 (4.33)	4.68 (3.16)	<0.001

*SD, standard deviation; SOFAS, social and occupational functioning assessment scale; QIDS, quick inventory of depressive symptomatology*.

* *t-test or chi-square*.

## Limitations and Future Directions

The CHARMS can be criticized for still relying on existing categories, criteria sets and diagnostic outcomes, even if in a “merged” form. However, in the absence of any valid alternatives, the CHARMS approach provides a workable solution, and is directly implementable in clinical practice. A future thought to entertain is the possibility that findings born out of HiTOP and RDoC frameworks might help to re-define the CHARMS criteria completely independent of DSM criteria sets and cut-offs.

Furthermore, the CHARMS pilot study has three assessment points only, which contrasts with the discussion earlier regarding the dynamic, fluctuating nature of emerging psychopathology, for which more frequent assessment points would be required in the future. However, studies are underway implementing more dynamic approaches in this cohort, including a combination of intensive longitudinal Ecological Momentary Assessment (EMA) for the duration of 4 months, paired with passive monitoring of sleep-wake cycles, and physical activity. These data-rich, fine-grained time series in this unique CHARMS-cohort will allow for the dynamic modeling of the onset of mental illness (see below).

### Dynamic Systems Approach and Joint Modeling

One approach we are taking is to conceptualize mental health as a complex dynamic system (99): A system composed of many elements which interact with each other over time. Other examples of complex systems comprise financial markets, the ecosystem of a lake, or the climate. While these systems are very diverse, they share underlying common (mathematical) principles which are universal to complex systems and describe their behavior. For example, the resilience of a system can be inferred by studying its stability, i.e., how far is a system from a phase transition or “tipping point” (100–102). In our CHARMS identification framework or clinical staging, a tipping point might represent the transition from subthreshold to threshold psychopathology, i.e., from at-risk mental state to full disorder (103, 104). The proximity of such phase transitions can be studied by resilience indicators or “early warning signs” identified in time series (e.g., time series of symptoms obtained using EMA) (105, 106).

Of relevance in the “dynamic” context is also our group's advancement of *joint modeling* in the mental health field. Joint modeling is a statistical technique that examines the dynamic association between variables assessed repeatedly over time (“longitudinal data”) and time-dependent outcomes (e.g., death, transition to psychosis). It represents a *joint* approach of combining multilevel models with random effects and a survival model. Our group has investigated the performance of joint modeling and dynamic prediction in the context of transition to psychosis in UHR individuals (107–109). We have shown that, compared to with traditional prediction models that rely exclusively on baseline data, joint modeling offers a superior approach in predicting psychosis in UHR individuals (107–109). Therefore, the joint modeling approach will also be explored in the context of dynamic prediction in our CHARMS cohort.

A further limitation of the CHARMS framework at this stage is the focus on four specific outcomes/at risk groups. Based on the outcomes of our (pilot) CHARMS cohort study, we will investigate the expansion of the framework to encompass other syndromes as well, such as obsessive compulsive disorders and eating disorders. These syndromes are captured and may in the future be incorporated as input and outcomes of interest.

A final criticism that the CHARMS framework faces is its reliance on clinical interview data only at this stage. Incorporating other (neurocognitive, bio-physiological) modalities will be an important next step, as there is a clear demand to identify markers of illness which directly map onto pathophysiology (110). Clinical staging enables the identification and evaluation of pathophysiology and biomarkers at each stage of illness (110). If we investigate the association between (bio)marker and symptoms in relation to stages in the CHARMS approach, we might be able to identify and differentiate between transdiagnostic markers as well as syndrome-specific markers. Future expansions of the study will include the incorporation of a neurocognitive test battery, blood-based biomarkers, and digital phenotyping.

## Conclusion

Emerging mental disorders develop in complex interacting trajectories over time with non-specific symptoms that overlap, intensify and recede, defying diagnostic borders. A new diagnostic approach and case identification framework is needed, with an emphasis on clinical utility. The clinical staging and the transdiagnostic CHARMS risk identification framework is guided by the principle that diagnostic terms or labels only need as much specificity to guide treatment selection (111). One key implication of the pluripotential CHARMS at-risk approach is that if identification and intervention can occur early, progression to later stages might be prevented. The instruments for such preventive treatment approaches, focusing on novel, broader target treatments such as psychosocial and neuroprotective approaches, will be trialed in this broad at-risk population.

## Data Availability Statement

The raw data supporting the conclusions of this article will be made available by the authors, without undue reservation.

## Ethics Statement

The studies involving human participants were reviewed and approved by Melbourne Health Ethics Committee. The patients/participants provided their written informed consent to participate in this study.

## Author Contributions

JH and BN developed the first draft. LD wrote sections of the manuscript. PM, GA, AC, CD, RG, AP, AR, and HY provided significant input to all sections of the manuscript. All authors approved the final manuscript.

## Conflict of Interest

PM reported receiving grant funding from National Alliance for Research on Schizophrenia and Depression and unrestricted research funding from AstraZeneca, Eli Lilly, Janssen-Cilag, Pfizer, and Novartis, as well as honoraria for educational activities with AstraZeneca, Eli Lilly, Janssen-Cilag, Pfizer, Bristol-Myers Squibb, Roche, and the Lundbeck Institute. The remaining authors declare that the research was conducted in the absence of any commercial or financial relationships that could be construed as a potential conflict of interest.

## Footnotes

^1^The UHR criteria are assessed using the Comprehensive Assessment of At-Risk Mental States (CAARMS, (19)). Young people at UHR are identified by one or more of the following characteristics: (1) Attenuated Psychotic Symptoms (APS)—young people who have experienced subthreshold, attenuated forms of positive psychotic symptoms during the past year, (2) Brief Limited Intermittent Psychotic Symptoms (BLIPS)—young people who have experienced episodes of frank psychotic symptoms that have not lasted longer than a week and have spontaneously abated, and (3) Trait and State Risk Factor (Trait)—individuals who have a first-degree relative with a psychotic disorder or who have a schizotypal personality disorder in addition to a significant decrease in functioning, or chronic low functioning, during the previous year.

## References

[1] Kessler RC Berglund P Demler O Jin R Merikangas KR Walters EE. Lifetime prevalence and age-of-onset distributions of DSM-IV disorders in the National Comorbidity Survey Replication. Arch Gen Psychiatry. (2005) 62:593–602. 10.1001/archpsyc.62.6.593 15939837

[2] Jones PB. Adult mental health disorders and their age at onset. Br J Psychiatry Suppl. (2013) 54:s5–10. 10.1192/bjp.bp.112.119164 23288502

[3] McGorry PD Killackey E Yung A. Early intervention in psychosis: concepts, evidence and future directions. World Psychiatry. (2008) 7:148–56. 10.1002/j.2051-5545.2008.tb00182.x 18836582 PMC2559918

[4] McGorry PD. Risk syndromes, clinical staging and DSM V: new diagnostic infrastructure for early intervention in psychiatry. Schizophr Res. (2010) 120:49–53. 10.1016/j.schres.2010.03.016 20456923

[5] Mei C Nelson B Hartmann J Spooner R McGorry PD Chapter 4 - Transdiagnostic early intervention, prevention, and prediction in psychiatry. In: Baune BT, editor. Personalized Psychiatry. San Diego: Academic Press, 27–37.

[6] McGorry PD Hickie IB Yung AR Pantelis C Jackson HJ. Clinical staging of psychiatric disorders: a heuristic framework for choosing earlier, safer and more effective interventions. Aust N Z J Psychiatry. (2006) 40:616–22. 10.1080/j.1440-1614.2006.01860.x 16866756

[7] McGorry P. Transition to adulthood: the critical period for pre-emptive, disease-modifying care for schizophrenia and related disorders. Schizophr Bull. (2011) 37:524–30. 10.1093/schbul/sbr027 21505119 PMC3080696

[8] Kessler RC Birnbaum H Demler O Falloon IR Gagnon E Guyer M. The prevalence and correlates of nonaffective psychosis in the National Comorbidity Survey Replication (NCS-R). Biol Psychiatry. (2005) 58:668–76. 10.1016/j.biopsych.2005.04.034 16023620 PMC2847859

[9] Kessler RC Ormel J Petukhova M McLaughlin KA Green JG Russo LJ. Development of lifetime comorbidity in the World Health Organization world mental health surveys. Arch Gen Psychiatry. (2011) 68:90–100. 10.1001/archgenpsychiatry.2010.180 21199968 PMC3057480

[10] Merikangas KR Herrell R Swendsen J Rossler W Ajdacic-Gross V Angst J. Specificity of bipolar spectrum conditions in the comorbidity of mood and substance use disorders: results from the Zurich cohort study. Arch Gen Psychiatry. (2008) 65:47–52. 10.1001/archgenpsychiatry.2007.18 18180428

[11] Merikangas KR He JP Burstein M Swanson SA Avenevoli S Cui L. Lifetime prevalence of mental disorders in US adolescents: results from the National Comorbidity Survey Replication–Adolescent Supplement (NCS-A). J Am Acad Child Adolesc Psychiatry. (2010) 49:980–9. 10.1016/j.jaac.2010.05.017 20855043 PMC2946114

[12] Merikangas KR Cui L Kattan G Carlson GA Youngstrom EA Angst J. Mania with and without depression in a community sample of US adolescents. Arch Gen Psychiatry. (2012) 69:943–51. 10.1001/archgenpsychiatry.2012.38 22566563 PMC11955849

[13] Murray GK Jones PB. Psychotic symptoms in young people without psychotic illness: mechanisms and meaning. Br J Psychiatry. (2012) 201:4–6. 10.1192/bjp.bp.111.107789 22753849

[14] Ormel J Raven D van Oort F Hartman CA Reijneveld SA Veenstra R. Mental health in Dutch adolescents: a TRAILS report on prevalence, severity, age of onset, continuity and co-morbidity of DSM disorders. Psychol Med. (2015) 45:345–60. 10.1017/S0033291714001469 25066533

[15] Cramer AO Waldorp LJ van der Maas HL Borsboom D. Comorbidity: a network perspective. Behav Brain Sci. (2010) 33:137–50; discussion 150–93. 10.1017/S0140525X09991567 20584369

[16] Borsboom D Cramer AO. Network analysis: an integrative approach to the structure of psychopathology. Annu Rev Clin Psychol. (2013) 9:91–121. 10.1146/annurev-clinpsy-050212-185608 23537483

[17] Borsboom D. A network theory of mental disorders. World Psychiatry. (2017) 16:5–13. 10.1002/wps.20375 28127906 PMC5269502

[18] Yung AR McGorry PD. The prodromal phase of first-episode psychosis: past and current conceptualizations. Schizophr Bull. (1996) 22:353–70. 10.1093/schbul/22.2.353 8782291

[19] Yung AR Yuen HP McGorry PD Phillips LJ Kelly D Dell'Olio M. Mapping the onset of psychosis: the Comprehensive Assessment of At-Risk Mental States. Aust N Z J Psychiatry. (2005) 39:964–71. 10.1080/j.1440-1614.2005.01714.x 16343296

[20] Malla AK Norman RM. Prodromal symptoms in schizophrenia. Br J Psychiatry. (1994) 164:487–93. 10.1192/bjp.164.4.487 8038937

[21] McGorry PD Mei C. Ultra-high-risk paradigm: lessons learnt and new directions. Evid Based Ment Health. (2018) 21:131–3. 10.1136/ebmental-2018-300061 30355661 PMC10270365

[22] Lin A Wood SJ Nelson B Beavan A McGorry P Yung AR. Outcomes of nontransitioned cases in a sample at ultra-high risk for psychosis. Am J Psychiatry. (2015) 172:249–58. 10.1176/appi.ajp.2014.13030418 25727537

[23] Rutigliano G Valmaggia L Landi P Frascarelli M Cappucciati M Sear V. Persistence or recurrence of non-psychotic comorbid mental disorders associated with 6-year poor functional outcomes in patients at ultra high risk for psychosis. J Affect Disord. (2016) 203:101–10. 10.1016/j.jad.2016.05.053 27285723

[24] Beck K Andreou C Studerus E Heitz U Ittig S Leanza L. Clinical and functional long-term outcome of patients at clinical high risk (CHR) for psychosis without transition to psychosis: a systematic review. Schizophr Res. (2019) 210:39–47. 10.1016/j.schres.2018.12.047 30651204

[25] Ajnakina O Morgan C Gayer-Anderson C Oduola S Bourque F Bramley S. Only a small proportion of patients with first episode psychosis come via prodromal services: a retrospective survey of a large UK mental health programme. BMC Psychiatry. (2017) 17:308. 10.1186/s12888-017-1468-y 28841826 PMC5574213

[26] Fusar-Poli P Bonoldi I Yung AR Borgwardt S Kempton MJ Valmaggia L. Predicting psychosis: meta-analysis of transition outcomes in individuals at high clinical risk. Arch Gen Psychiatry. (2012) 69:220–9. 10.1001/archgenpsychiatry.2011.1472 22393215

[27] Cuijpers P. Examining the effects of prevention programs on the incidence of new cases of mental disorders: the lack of statistical power. Am J Psychiatry. (2003) 160:1385–91. 10.1176/appi.ajp.160.8.1385 12900296

[28] Shankman SA Lewinsohn PM Klein DN Small JW Seeley JR Altman SE. Subthreshold conditions as precursors for full syndrome disorders: a 15-year longitudinal study of multiple diagnostic classes. J Child Psychol Psychiatry. (2009) 50:1485–94. 10.1111/j.1469-7610.2009.02117.x 19573034 PMC2804772

[29] Krueger RF Piasecki TM. Toward a dimensional and psychometrically-informed approach to conceptualizing psychopathology. Behav Res Ther. (2002) 40:485–99. 10.1016/S0005-7967(02)00016-5 12038642

[30] Krueger RF Markon KE. A dimensional-spectrum model of psychopathology: progress and opportunities. Arch Gen Psychiatry. (2011) 68:10–1. 10.1001/archgenpsychiatry.2010.188 21199961

[31] Johns LC van Os J. The continuity of psychotic experiences in the general population. Clin Psychol Rev. (2001) 21:1125–41. 10.1016/S0272-7358(01)00103-9 11702510

[32] van Os J Linscott RJ Myin-Germeys I Delespaul P Krabbendam L. A systematic review and meta-analysis of the psychosis continuum: evidence for a psychosis proneness-persistence-impairment model of psychotic disorder. Psychol Med. (2009) 39:179–95. 10.1017/S0033291708003814 18606047

[33] Fleming S Shevlin M Murphy J Joseph S Psychosis within dimensional and categorical models of mental illness. Psychosis. (2013) 6:4–15. 10.1080/17522439.2012.752027

[34] Linscott RJ van Os J. An updated and conservative systematic review and meta-analysis of epidemiological evidence on psychotic experiences in children and adults: on the pathway from proneness to persistence to dimensional expression across mental disorders. Psychol Med. (2013) 43:1133–49. 10.1017/S0033291712001626 22850401

[35] Pickles A Angold A. Natural categories or fundamental dimensions: on carving nature at the joints and the rearticulation of psychopathology. Dev Psychopathol. (2003) 15:529–51. 10.1017/S0954579403000282 14582931

[36] Widiger TA Samuel DB Diagnostic categories or dimensions? A question for the Diagnostic And Statistical Manual Of Mental Disorders–fifth edition J Abnorm Psychol. (2005) 114:494–504. 10.1037/0021-843X.114.4.494 16351373

[37] McGorry PD. Issues for DSM-V: clinical staging: a heuristic pathway to valid nosology and safer, more effective treatment in psychiatry. Am J Psychiatry. (2007) 164:859–60. 10.1176/ajp.2007.164.6.859 17541042

[38] Scott J Leboyer M Hickie I Berk M Kapczinski F Frank E. Clinical staging in psychiatry: a cross-cutting model of diagnosis with heuristic and practical value. Br J Psychiatry. (2013) 202:243–5. 10.1192/bjp.bp.112.110858 23549937

[39] Hickie IB Scott EM Hermens DF Naismith SL Guastella AJ Kaur M. Applying clinical staging to young people who present for mental health care. Early Interv Psychiatry. (2013) 7:31–43. 10.1111/j.1751-7893.2012.00366.x 22672533

[40] Cross SP Hermens DF Scott EM Ottavio A McGorry PD Hickie IB. A clinical staging model for early intervention youth mental health services. Psychiatr Serv. (2014) 65:939–43. 10.1176/appi.ps.201300221 24828746

[41] Fusar-Poli P McGorry PD Kane JM. Improving outcomes of first-episode psychosis: an overview. World Psychiatry. (2017) 16:251–65. 10.1002/wps.20446 28941089 PMC5608829

[42] Iorfino F Scott EM Carpenter JS Cross SP Hermens DF Killedar M. Clinical stage transitions in persons aged 12 to 25 years presenting to early intervention mental health services with anxiety, mood, and psychotic disorders. JAMA Psychiatry. (2019) 76:1167–75. 10.1001/jamapsychiatry.2019.2360 31461129 PMC6714017

[43] Cross SPM Scott J Hickie IB. Predicting early transition from sub-syndromal presentations to major mental disorders. BJPsych Open. (2017) 3:223–7. 10.1192/bjpo.bp.117.004721 28959452 PMC5596309

[44] McGorry P Nelson B. Why we need a transdiagnostic staging approach to emerging psychopathology, early diagnosis, and treatment. JAMA Psychiatry. (2016) 73:191–2. 10.1001/jamapsychiatry.2015.2868 26765254

[45] McGorry PD Hartmann JA Spooner R Nelson B. Beyond the “at risk mental state” concept: transitioning to transdiagnostic psychiatry. World Psychiatry. (2018) 17:133–42. 10.1002/wps.20514 29856558 PMC5980504

[46] Hartmann JA Nelson B Spooner R Paul Amminger G Chanen A Davey CG. Broad clinical high-risk mental state (CHARMS): Methodology of a cohort study validating criteria for pluripotent risk. Early Interv Psychiatry. (2019) 13:379–86. 10.1111/eip.12483 28984077

[47] Spooner R Hartmann JA McGorry PD Nelson B Chapter 21 - New paradigms to study psychosis risk: clinical staging, pluripotency, and dynamic prediction. In: Thompson AD Broome MR, editors. Risk Factors for Psychosis. Academic Press, 399–416.

[48] Chanen AM Berk M Thompson KJH. Integrating early intervention for borderline personality disorder and mood disorders. Harv Rev Psychiatry. (2016) 24:330–41. 10.1097/HRP.0000000000000105 27144298

[49] Mazer AK Cleare AJ Young AH Juruena MF. Bipolar affective disorder and borderline personality disorder: differentiation based on the history of early life stress and psychoneuroendocrine measures. Behav Brain Res. (2019) 357:48–56. 10.1016/j.bbr.2018.04.015 29702176

[50] Cavelti M Thompson K Chanen A Kaess M. Psychotic symptoms in borderline personality disorder: developmental aspects. Curr Opin Psychol. (2020) 37:26–31. 10.1016/j.copsyc.2020.07.003 32771980

[51] Johannessen JO McGorry P DSM-5 and the ‘Psychosis Risk Syndrome’: the need for a broader perspective. Psychosis. (2010) 2:93–6. 10.1080/17522431003759974

[52] Fusar-Poli P Carpenter WT Woods SW McGlashan TH Attenuated psychosis syndrome: ready for DSM-5.1? Annu Rev Clin Psychol. (2014) 10:155–92. 10.1146/annurev-clinpsy-032813-153645 24471375

[53] Fusar-Poli P The hype cycle of the clinical high risk state for psychosis: the need of a refined approach. Schizophr Bull. (2018) 44:250–3. 10.1093/schbul/sbx181 PMC5216870 28053129

[54] Lee TY Lee J Kim M Choe E Kwon JS. Can we predict psychosis outside the clinical high-risk state? A systematic review of non-psychotic risk syndromes for mental disorders. Schizophr Bull. (2018) 44:276–85. 10.1093/schbul/sbx173 29438561 PMC5814842

[55] Woods SW Powers AR 3rd Taylor JH Davidson CA Johannesen JK Addington J. Lack of diagnostic pluripotentiality in patients at clinical high risk for psychosis: specificity of comorbidity persistence and search for pluripotential subgroups. Schizophr Bull. (2018) 44:254–63. 10.1093/schbul/sbx138 29036402 PMC5814797

[56] Amos AJ. A review of spin and bias use in the early intervention in psychosis literature. Prim Care Companion CNS Disord. (2014) 16:P 10.4088/PCC.13r01586 24940528 PMC4048144

[57] Goodday SM Duffy A. Shedding light on the onset of psychiatric illness: looking through a developmental lens. Evid Based Ment Health. (2019) 22:134–6. 10.1136/ebmental-2018-300076 30665990 PMC10270460

[58] McGorry P van Os J. Redeeming diagnosis in psychiatry: timing versus specificity. Lancet. (2013) 381:343–5. 10.1016/S0140-6736(12)61268-9 23351805

[59] Patalay P Fonagy P Deighton J Belsky J Vostanis P Wolpert M. A general psychopathology factor in early adolescence. Br J Psychiatry. (2015) 207:15–22. 10.1192/bjp.bp.114.149591 25906794

[60] Murray AL Eisner M Ribeaud D. The development of the general factor of psychopathology 'p factor' through childhood and adolescence. J Abnorm Child Psychol. (2016) 44:1573–86. 10.1007/s10802-016-0132-1 26846993

[61] McElroy E Belsky J Carragher N Fearon P Patalay P Developmental stability of general and specific factors of psychopathology from early childhood to adolescence: dynamic mutualism or p-differentiation? J Child Psychol Psychiatry. (2018) 59:667–75. 10.1111/jcpp.12849 29197107 PMC6001631

[62] Wigman JT van Os J Thiery E Derom C Collip D Jacobs N. Psychiatric diagnosis revisited: towards a system of staging and profiling combining nomothetic and idiographic parameters of momentary mental states. PLoS One. (2013) 8:e59559. 10.1371/journal.pone.0059559 23555706 PMC3610753

[63] Boschloo L van Borkulo CD Rhemtulla M Keyes KM Borsboom D Schoevers RA. The network structure of symptoms of the diagnostic and statistical manual of mental disorders. PLoS One. (2015) 10:e0137621. 10.1371/journal.pone.0137621 26368008 PMC4569413

[64] Pe ML Kircanski K Thompson RJ Bringmann LF Tuerlinckx F Mestdagh M. Emotion-network density in major depressive disorder. Clin Psychol Sci. (2015) 3:292–300. 10.1177/2167702614540645 31754552 PMC6871506

[65] Boschloo L Schoevers RA van Borkulo CD Borsboom D Oldehinkel AJ. The network structure of psychopathology in a community sample of preadolescents. J Abnorm Psychol. (2016) 125:599–606. 10.1037/abn0000150 27030994

[66] Groen RN Wichers M Wigman JTW Hartman CA. Specificity of psychopathology across levels of severity: a transdiagnostic network analysis. Sci Rep. (2019) 9:18298. 10.1038/s41598-019-54801-y 31797974 PMC6892855

[67] Kim-Cohen J Caspi A Moffitt TE Harrington H Milne BJ Poulton R. Prior juvenile diagnoses in adults with mental disorder: developmental follow-back of a prospective-longitudinal cohort. Arch Gen Psychiatry. (2003) 60:709–17. 10.1001/archpsyc.60.7.709 12860775

[68] Patton GC Coffey C Romaniuk H Mackinnon A Carlin JB Degenhardt L. The prognosis of common mental disorders in adolescents: a 14-year prospective cohort study. Lancet. (2014) 383:1404–11. 10.1016/S0140-6736(13)62116-9 24439298

[69] Hartmann JA Nelson B Ratheesh A Treen D McGorry PD. At-risk studies and clinical antecedents of psychosis, bipolar disorder and depression: a scoping review in the context of clinical staging. Psychol Med. (2019) 49:177–89. 10.1017/S0033291718001435 29860956

[70] Tsuang MT Bar JL Stone WS Faraone SV. Gene-environment interactions in mental disorders. World Psychiatry. (2004) 3:73–83. 16633461 PMC1414673

[71] Lichtenstein P Yip BH Bjork C Pawitan Y Cannon TD Sullivan PF. Common genetic determinants of schizophrenia and bipolar disorder in Swedish families: a population-based study. Lancet. (2009) 373:234–9. 10.1016/S0140-6736(09)60072-6 19150704 PMC3879718

[72] Agius M Goh C Ulhaq S McGorry P. The staging model in schizophrenia, and its clinical implications. Psychiatr Danub. (2010) 22:211–20. 20562749

[73] Anttila V. Bulik-Sullivan B. Finucane H. K. Walters R. K. Bras J. Duncan L.. (2018). Analysis of shared heritability in common disorders of the brain. Science 360:eaap8757. 10.1126/science.aap8757 29930110 PMC6097237

[74] Mansell W. Transdiagnostic psychiatry goes above and beyond classification. World Psychiatry. (2019) 18:360–1. 10.1002/wps.20680 31496093 PMC6732685

[75] Duffy A Malhi GS. Mapping the developmental trajectory of bipolar disorder: Importance of prerequisite groundwork. Aust N Z J Psychiatry. (2017) 51:761–3. 10.1177/0004867417720035 28718721

[76] Scott J Henry C Clinical staging models: from general medicine to mental disorders. BJPsych Adv. (2017) 23:292–9. 10.1192/apt.bp.116.016436

[77] Insel T Cuthbert B Garvey M Heinssen R Pine DS Quinn K Research domain criteria (RDoC): toward a new classification framework for research on mental disorders. Am J Psychiatry. (2010) 167:748–51. 10.1176/appi.ajp.2010.09091379 20595427

[78] Cuthbert BN Insel TR. Toward the future of psychiatric diagnosis: the seven pillars of RDoC. BMC Med. (2013) 11:126. 10.1186/1741-7015-11-126 23672542 PMC3653747

[79] Kozak MJ Cuthbert BN. The NIMH research domain criteria initiative: background, issues, and pragmatics. Psychophysiology. (2016) 53:286–97. 10.1111/psyp.12518 26877115

[80] Kotov R Krueger RF Watson D Achenbach TM Althoff RR Bagby RM. The Hierarchical Taxonomy of Psychopathology (HiTOP): a dimensional alternative to traditional nosologies. J Abnorm Psychol. (2017) 126:454–77. 10.31234/osf.io/zaadn 28333488

[81] Kotov R Krueger RF Watson D. A paradigm shift in psychiatric classification: the Hierarchical Taxonomy Of Psychopathology (HiTOP). World Psychiatry. (2018) 17:24–5. 10.1002/wps.20478 29352543 PMC5775140

[82] Kotov R Jonas KG Carpenter WT Dretsch MN Eaton NR Forbes MK. Validity and utility of Hierarchical Taxonomy of Psychopathology (HiTOP): I. Psychosis superspectrum. World Psychiatry. (2020) 19:151–72. 10.1002/wps.20730 32394571 PMC7214958

[83] van Os J Linscott RJ. Introduction: the extended psychosis phenotype–relationship with schizophrenia and with ultrahigh risk status for psychosis. Schizophr Bull. (2012) 38:227–30. 10.1093/schbul/sbr188 22355185 PMC3283160

[84] Widiger TA Sellbom M Chmielewski M Clark LA DeYoung CG Kotov R Personality in a Hierarchical model of psychopathology. Clin Psychol Sci. (2019) 7:77–92. 10.1177/2167702618797105

[85] Krueger RF Watson D Widiger TA The vibrant intersection of personality and psychopathology research: a special issue of the *Journal of Research in Personality*. J. Res Pers. (2020) 84:103890 10.1016/j.jrp.2019.103890

[86] Sharp C Wright AG Fowler JC Frueh BC Allen JG Oldham J. The structure of personality pathology: Both general ('g') and specific ('s') factors? J Abnorm Psychol. (2015) 124:387–98. 10.1037/abn0000033 25730515

[87] Thompson KN Jackson H Cavelti M Betts J McCutcheon L Jovev M. The clinical significance of subthreshold borderline personality disorder features in outpatient youth. J Pers Disord. (2019) 33:71–81. 10.1521/pedi_2018_32_330 30036169

[88] Duffy A Goodday S Passos IC Kapczinski F. Changing the bipolar illness trajectory. Lancet Psychiatry. (2017) 4:11–3. 10.1016/S2215-0366(16)30352-2 28012467

[89] Angold A Costello EJ Erkanli A. Comorbidity. J Child Psychol Psychiatry. (1999) 40:57–87. 10.1111/1469-7610.00424 10102726

[90] Burke JD Loeber R Lahey BB Rathouz PJ. Developmental transitions among affective and behavioral disorders in adolescent boys. J Child Psychol Psychiatry. (2005) 46:1200–10. 10.1111/j.1469-7610.2005.00422.x 16238667

[91] Kessler RC Avenevoli S McLaughlin KA Green JG Lakoma MD Petukhova M. Lifetime co-morbidity of DSM-IV disorders in the US National Comorbidity Survey Replication Adolescent Supplement (NCS-A). Psychol Med. (2012) 42:1997–2010. 10.1017/S0033291712000025 22273480 PMC3448706

[92] Shevlin M McElroy E Murphy J. Homotypic and heterotypic psychopathological continuity: a child cohort study. Soc Psychiatry Psychiatr Epidemiol. (2017) 52:1135–45. 10.1007/s00127-017-1396-7 28550520 PMC5581823

[93] Lahey BB Zald DH Hakes JK Krueger RF Rathouz PJ. Patterns of heterotypic continuity associated with the cross-sectional correlational structure of prevalent mental disorders in adults. JAMA Psychiatry. (2014) 71:989–96. 10.1001/jamapsychiatry.2014.359 24989054 PMC4160409

[94] Plana-Ripoll O Pedersen CB Holtz Y Benros ME Dalsgaard S de Jonge P. Exploring comorbidity within mental disorders among a Danish national population. JAMA Psychiatry. (2019) 76:259–70. 10.1001/jamapsychiatry.2018.3658 30649197 PMC6439836

[95] Caspi A Houts RM Ambler A Danese A Elliott ML Hariri A Longitudinal assessment of mental health disorders and comorbidities across 4 decades among participants in the Dunedin birth cohort Study. JAMA Netw Open. (2020) 3:e203221 10.1001/jamanetworkopen.2020.3221 32315069 PMC7175086

[96] Krueger RF Eaton NR Transdiagnostic factors of mental disorders. World Psychiatry. (2015) 14:27–9. 10.1002/wps.20175 25655146 PMC4329885

[97] van Os J Guloksuz S. A critique of the “ultra-high risk” and “transition” paradigm. World Psychiatry. (2017) 16:200–6. 10.1002/wps.20423 28498576 PMC5428198

[98] Guloksuz S van Os J. Need for evidence-based early intervention programmes: a public health perspective. Evid Based Ment Health. (2018) 21:128–30. 10.1136/ebmental-2018-300030 30282627 PMC10270415

[99] Nelson B McGorry PD Wichers M Wigman JTW Hartmann JA. Moving from static to dynamic models of the onset of mental disorder: a review. JAMA Psychiatry. (2017) 74:528–34. 10.1001/jamapsychiatry.2017.0001 28355471

[100] Scheffer M Critical Transitions in Nature and Society. Princeton: Princeton University Press (2009).

[101] Scheffer M Bascompte J Brock WA Brovkin V Carpenter SR Dakos V. Early-warning signals for critical transitions. Nature. (2009) 461:53–9. 10.1038/nature08227 19727193

[102] Scheffer M Carpenter SR Lenton TM Bascompte J Brock W Dakos V. Anticipating critical transitions. Science. (2012) 338:344–8. 10.1126/science.1225244 23087241

[103] Hartmann J Wichers M McGorry P Nelson B Tipping points:-predicting transitions to mental illness and remission in at-risk young people. Early Interv Psychiatry. (2018) 12:12–12.

[104] Hartmann JA McGorry PD Wichers M Nelson B T30. Tipping POINTS-PREDICTING TRANSITIONS TO PSYCHOSIS IN AT-RISK YOUNG PEOPLE *Schizophr Bull*. (2018) 44:S124–S5. 10.1093/schbul/sby016.306

[105] van de Leemput IA Wichers M Cramer AO Borsboom D Tuerlinckx F Kuppens P. Critical slowing down as early warning for the onset and termination of depression. Proc Natl Acad Sci U S A. (2014) 111:87–92. 10.1073/pnas.1312114110 24324144 PMC3890822

[106] Wichers M Groot PC Psychosystems ESMGEWSG. Critical slowing down as a personalized early warning signal for depression. Psychother Psychosom. (2016) 85:114–6. 10.1159/000441458 26821231

[107] Yuen HP Mackinnon A Hartmann J Amminger GP Markulev C Lavoie S. Dynamic prediction of transition to psychosis using joint modelling. Schizophr Res. (2018) 202:333–40. 10.1016/j.schres.2018.07.002 30539771

[108] Yuen HP Mackinnon A Nelson B. A new method for analysing transition to psychosis: Joint modelling of time-to-event outcome with time-dependent predictors. Int J Methods Psychiatr Res (2018) 27:e1588. 10.1002/mpr.1588 28944523 PMC6877213

[109] Yuen HP Mackinnon A Nelson B. Dynamic prediction systems of transition to psychosis using joint modelling: extensions to the base system. Schizophr Res. (2020) 216:207–12. 10.1016/j.schres.2019.11.059 31839554

[110] McGorry P Keshavan M Goldstone S Amminger P Allott K Berk M Biomarkers and clinical staging in psychiatry. World Psychiatry. (2014) 13:211–23. 10.1002/wps.20144 25273285 PMC4219053

[111] McGorry PD Diagnosis without borders: a pluripotential approach to preventive intervention in emerging mental disorders. In Clinical Staging in Psychiatry: Making Diagnosis Work for Research and Treatment. McGorry PD Hickie IB, editors. Cambridge: Cambridge University Press (2019), p. 1–11. 10.1017/9781139839518.001

